# Supplementary material for: Nitrogen dynamics as a function of soil types, compaction, and moisture
Source: PLoS One. 2024 Apr 4;19(4):e0301296. doi: 10.1371/journal.pone.0301296 (PMC10996285; doi:10.1371/journal.pone.0301296)
Supplement: S1 Table — (DOCX) [file pone.0301296.s001.docx]

**Table 1** – Baseline properties of the soil used in the experiment

| **Properties (units)** | **Sandy Loam** | **Loam** |
| --- | --- | --- |
| Soil pH | 8.0 | 7.3 |
| Buffer pH | 7.2 | 7.2 |
| EC (mmho/cm) | 0.32 | 0.38 |
| Organic matter (g kg^-1^) | 22 | 43 |
| NO_3_−N (mg kg^-1^) | 39.7 | 63.5 |
| Olsen P (mg kg^-1^) | 17.8 | 52.6 |
| K (mg kg^-1^) | 697 | 1260 |
| SO_4_^2−^S (mg kg^-1^) | 40.3 | 10.6 |
| Zn (mg kg^-1^) | 1.07 | 2.44 |
| Fe (mg kg^-1^) | 5.2 | 18.9 |
| Mn (mg kg^-1^) | 4.4 | 13.8 |
| Cu (mg kg^-1^) | 0.58 | 0.63 |
| Ca (mg kg^-1^) | 3426 | 2640 |
| Mg (mg kg^-1^) | 348 | 246 |
| Na (mg kg^-1^) | 77 | 16 |
| B (mg kg^-1^) | 1.27 | 0.77 |
| CEC (meq/100g) | 22.1 | 18.6 |
| Organic Carbon (g kg^-1^) | 9.6 | 22.8 |
| Total Carbon (g kg^-1^) | 10.5 | 23.6 |
