# Supplementary material for: Nitrogen dynamics as a function of soil types, compaction, and moisture
Source: PLoS One. 2024 Apr 4;19(4):e0301296. doi: 10.1371/journal.pone.0301296 (PMC10996285; doi:10.1371/journal.pone.0301296)
Supplement: S2 Table — (DOCX) [file pone.0301296.s002.docx]

**Supplementary file S2**

Precipitation regime used for this study.

|  | Dry weather (mm) | Wet weather (mm) |
| --- | --- | --- |
| **Day** | **1981 - 2010** | **2019** |
| 1 | 2.032 | 3.81 |
| 2 | 1.778 | 0.254 |
| 3 | 1.778 | 0 |
| 4 | 2.032 | 0 |
| 5 | 1.778 | 0 |
| 6 | 1.778 | 0.762 |
| 7 | 1.524 | 0.762 |
| 8 | 1.778 | 4.318 |
| 9 | 1.524 | 1.778 |
| 10 | 1.524 | 4.826 |
| 11 | 1.524 | 0 |
| 12 | 1.778 | 0 |
| 13 | 1.778 | 0 |
| 14 | 1.524 | 0 |
| 15 | 1.778 | 0 |
| 16 | 1.524 | 0.762 |
| 17 | 1.778 | 0.254 |
| 18 | 2.032 | 6.858 |
| 19 | 1.778 | 0 |
| 20 | 1.778 | 1.27 |
| 21 | 2.286 | 26.924 |
| 22 | 2.286 | 12.954 |
| 23 | 2.54 | 0 |
| 24 | 2.286 | 0.508 |
| 25 | 2.54 | 0 |
| 26 | 2.54 | 0.762 |
| 27 | 2.54 | 1.778 |
| 28 | 3.048 | 19.304 |
| 29 | 3.302 | 40.64 |
| 30 | 3.302 | 0.254 |
| 31 | 3.556 | 0 |
