# Supplementary material for: Nitrogen dynamics as a function of soil types, compaction, and moisture
Source: PLoS One. 2024 Apr 4;19(4):e0301296. doi: 10.1371/journal.pone.0301296 (PMC10996285; doi:10.1371/journal.pone.0301296)
Supplement: Attachment — Submitted filename: Comments to the Author.pdf [file pone.0301296.s003.pdf]

Comments to the Author:

Line 17: I would put soil types (loam and sandy loam) in parentheses.

Line 34: Here 'moist soil' might be too broad, as you have only potentially shown this outcome in sandy loam and loam soil, not all types of soil.

Line 38: Lowercase O in word 'Oxide'.

Line 41: Remove space before paragraph. Keep format consistent throughout the manuscript.

Line 53-54: This sentence is partially true. Denitrification and volatilization are just one of the factors contributing global warming, as carbon dioxide accounting for a larger proportion.

Line 54-56: Add references.

Line 82-85: Add references explain how this subsequently affect N transformation and movement.

Line 71-100: The title of this manuscript is 'nitrogen dynamics as a function of soil types, compaction, and moisture'. Here only background information of compaction was introduced, it would be good if some background information of used soil types and moisture can be added.

Line 131: Compaction affects N dynamics. Can you explain how you pack sandy loam soil into columns with a targeted bulk density of 1.6 kg/m3 on the top 10 cm depth? Will the bulk density of bottom 10 cm depth soil be affected during this packing process?

Line 149-153: Water was added daily, please give an example of how much water content (in mL) was added per day?

Line 155: Lowercase C in word 'collection'. Remove space before paragraph.

Line 161: Can you explain how you determine the time interval and what is the threshold for each sponge to absorb ammonia?

Line 164-165: Lowercase K in word 'kg'.

Linr166-168: The volume of N2O gas for analysis and calculation is confusing. Please explain it and show the calculation.

Line 183: Check the hyphen in NH4-N and NO3-N.

Line 187: This section should be 2.4 not 2.5. Lowercase A in word 'Analysis'. Remove space before paragraph.

Line 217: lowercase A in word 'ammonia'.

Line 228: There's no difference in N2O emission among all treatments, except CxD treatment.

Line 238-239: 'both exhibited similar patterns of gas emissions' is confusing. This is not true for NH3 and N2O. Please explain it.

Line 240: The trend you described here is correct if it's NH3 volatilization value, but not for NH3 volatilization rate.

Line 246-247: Add space between paragraphs.

Line 257: The lowest was observed in wet weather under control treatment (here should be CxW, not CxD). The lowest was also observed in SCxW treatment, there's no difference in residual NH4-N between SCxW and CxW.

Line 259: 4 in 'NH4-N' should be the subscript.

Line 262: Lowercase K in word 'kg'.

Line 323: Change the reference format of BEAUCHAMP et al., 1982 and check other citations' format throughout the manuscript.

Line 324-325: Add references, how much initial soil moisture content will be considered as high?

Line 327-329: Will rain cause more fertilizer losses through surface runoff compared to reducing NH3 volatilization?

Line 344-346: Loam soil possesses clay particles……, sandy loam could have same amount of clay content, please specify the percentages of clay, silt, and sand for the two types of soil in this study.

Line 369: Remove comma.

Line 386-387: In the word 'NO3-N', 3 should be the subscript.

Line 387-389: Add references.

Line 441: For those soil with high organic matter and residual nitrate, why not suggest using less amount of fertilizer?

Figure 2: In the results part, you mentioned Figure 2A and 2B, alphabet ‘A’ and ‘B’ should also show in each figure.
Figure 3 and 4: Lowercase K in word ‘kg’.
Table 1: The standard deviations for leachate and soil residual mineral N are relatively high, so I’m wondering if this experiment repeatable or not?
Table 2: P value in this table is confusing, I would put p value at the bottom of each section instead of top. Symbol ‘±’ is missing in terms of residual NH4-N under SCxW treatment.
