# Supplementary material for: Nitrogen dynamics as a function of soil types, compaction, and moisture
Source: PLoS One. 2024 Apr 4;19(4):e0301296. doi: 10.1371/journal.pone.0301296 (PMC10996285; doi:10.1371/journal.pone.0301296)
Supplement: Attachment — Submitted filename: Reviewers comments.pdf [file pone.0301296.s004.pdf]

The authors have conducted the present study on "Nitrogen dynamics as a function of soil types, compaction, and moisture", which is of practical significance in view of the low use efficiency of N. The experiment has been designed well. A few comments are given below for further improvement.

Abstract: Authors have not studied the method of application (broadcasting/ split) of fertilizer in soil and its effect on N-dynamics in the present study. Hence, the statement in connection with the split application may be deleted.

Introduction:

The authors emphasized soil compaction and moisture content, while formulating the hypothesis. However, it does not emphasize soil types or textural composition.

Research gap is poorly defined.

The novelty in the experimental set up (simulated condition) in carrying out studies on N dynamics may be highlighted.

Materials and methods

USDA soil Taxonomy may be used against the soils used in the study.

Description of the climatic condition (data) of the sampling sites may be included in this section, since the sampling sites vary in climatic condition.

Details of application method (e.g. spray) of UAN in soil to be highlighted.

Why was the N dose equivalent to 200 kg N/ha applied? what is the basis?

The methodologies followed to characterize the soils for its properties may be included in this section. For the micronutrients, clearly indicate the extractant(s) used for the estimation of the nutrients in soil.

Potassium contents of soils are very high, the reason may be given. The Olsen P in loamy soil seems to be high. The data may be rechecked.

Results

Reason behind higher leaching of nitrate (NO_3^-) under loamy soil than sandy loam soil (with higher sand percentage) may be indicated.

Line no. 226-229: highest N_2O emission was recorded in control treatment in dry weather (C x D), and the effects of other treatments were at par with that in SSC x D. please rectify.

Residual nitrate was significantly lower in sandy loam soil…it indicates higher leaching in sandy loam (since mean effect of soil type on other losses were non-significant). But the data indicates the reverse.

Why under compacted condition, residual nitrate was reduced in-spite of the fact that nitrous oxide emission was significantly lower under compacted condition.

Rectify the line 331-333.

In case of control and surface compact condition under dry and wet situation, residual ammonium was reported lower in loamy soil in comparison to sandy loam soil. The author did not consider the cation exchange capacity (CEC) of soils. It is reported in the study that the CEC of sandy loam soil is higher in-spite of the fact that organic carbon content of the soil is lower than that of the loamy soil. Cation exchange capacity of soil has significant positive impact on the retention of positively charged ammonium ion (NH_4^+) in soil.

Recommendation paragraph

Authors have not recorded the soil or air temperature. Hence, the sentence in connection with soil and air temperature may be deleted from the recommendation paragraph.

The manuscript may be accepted for publication following major revision.
